# Evaluation of Cytotoxicity and Taste-Masking Effect of Selected Flavors on Dental Lidocaine HCl Injection

**DOI:** 10.3390/ph13110353

**Published:** 2020-10-29

**Authors:** Sai H. S. Boddu, Diwakar B. Tukaramrao, Moawia M. Al-Tabakha, Akram Ashames, Rabin Neupane, R. Jayachandra Babu, Jwala Renukuntla, Amit K. Tiwari

**Affiliations:** 1 Department of Pharmaceutical Sciences, College of Pharmacy and Health Sciences, Ajman University, P.O. Box 346, Ajman, UAE; m.altabakha@ajman.ac.ae (M.M.A.-T.); a.ashames@ajman.ac.ae (A.A.); 2 Department of Pharmacology and Experimental Therapeutics, The University of Toledo, Health Science Campus, 3000 Arlington Ave, Toledo, OH 43614, USA; Diwakar.Tukaramrao@UToledo.Edu (D.B.T.); Rabin.Neupane@rockets.utoledo.edu (R.N.); Amit.Tiwari@UToledo.edu (A.K.T.); 3 Department of Drug Discovery and Development, Harrison School of Pharmacy, Auburn University, Auburn, AL 36849, USA; ramapjb@auburn.edu; 4 Department of Basic Pharmaceutical Sciences, Fred Wilson School of Pharmacy, High Point University, High Point, NC 27268, USA; jrenukun@highpoint.edu

**Keywords:** taste-masking, flavors, sweeteners, lidocaine HCl injection, dental anesthetics

## Abstract

Aim: Anxiety and intolerance to dental local anesthetic injections are common in patients undergoing dental procedures. This work was designed to study cytotoxicity of selected flavors in primary gingival keratinocytes (PGK), to acquire information on their suitability for use in dental lidocaine hydrochloride (LID) injection. We also evaluated the bio-mimetic taste of LID dental injection in the presence of selected flavors and sweetener using an Astree electronic tongue (ETongue). Methods: The cytotoxicity of chocolate natural and artificial flavor (CTE), raspberry flavor artificial (RAS), cherry flavor (CHR), bitterness suppressor flavor (BSF) and lemon flavor extract (LFE) at various dilutions (0.16–10% *v*/*v*) was carried out in PGK using the live cell morphological analysis and MTT cell cytotoxicity assay. Based on the cytotoxicity data, CTE and RAS were added to Xylocaine^®^ (2%) along with 0.09% sodium saccharin and taste was assessed using an ETongue. Results: After three hours of treatment, a dose-dependent cell death was induced by all flavors compared to the untreated control. BSF was found to be more toxic when compared to other flavors. CTE was found to be less toxic. The mean IC50 values of CTE, RAS, CHR, BSF and LFE in PGK were found to be 9.54, 8.43, 2.21, 0.38 and 4.01 mg/mL. Taste analysis with the ETongue showed a clear taste difference between the control and test formulations containing CTE and RAS flavors along with sodium saccharin. Conclusion: CTE and RAS flavors in combination with 0.09% sodium saccharin can achieve a significant taste-masking effect in the dental LID injection.

## 1. Introduction

“The bitter the taste of the drug, the better the cure” was a prevailing attitude of patients historically. However, this notion has changed, and patients now expect and demand oral medications to have a palatable taste and a pleasant flavor. Undesirable taste of drugs most often results in a lack of patient adherence and tolerance. Masking bitter taste of drugs is essential in order to ensure patient acceptability, especially with pediatric medications [1]. Lidocaine hydrochloride (LID), a fast-acting local anesthetic, acts by blocking certain functions of the nervous system and prevents transmission of pain impulses from the treated area to the brain. LID is known to efficiently control pain for a short duration when administered as an injection in the oral cavity. Xylocaine^®^, a 2% LID with epinephrine (1:100,000 or 1:50,000) is routinely used in dentistry to provide relief from pain during dental procedures [2]. Lidocaine has the ability to block sodium (Na^+^) channels in the nerve endings during initiation and conduction of nerve impulses. This prevents depolarization of neurons resulting in anesthetic effects of the drug. Epinephrine is essential for establishing satisfactory anesthesia by reducing systemic absorption. Over 300 million dental cartridges are injected every year in the United States and over 1.96 billion worldwide [3,4,5,6]. Onset time for pulpal anesthesia is around 5–10 min and continues for ~60 min with the vasoconstrictive effect of epinephrine. However, this injection has several drawbacks such as (i) low pH (~3.5, equivalent to pH of lemon juice) causing a stinging sensation upon injection, (ii) slow onset of action and (iii) unpalatable metallic taste and a bitter aftertaste [7,8]. These drawbacks are not well-received by patients in dentistry leading to poor medication tolerance [8]. As a result, providing a more conducive environment during the dental procedures requiring the use of local anesthetics has become challenging for dentists, especially in children.

In the literature, a wide variety of techniques such as the addition of sweeteners, complexing with cyclodextrins, coating with insoluble polymers [9], the use of ion-exchange resins [10], the addition of flavors [11] and the use of prodrugs [12] are reported for reducing bitter and unacceptable tastes of orally administered drugs [13]. However, these techniques are not accepted in an injectable formulation due to stringent requirements by the FDA. Taste-masking of injectables is generally not required as they are administered via subcutaneous, intravenous, intramuscular routes; however, this may not be applicable for injections made in the oral cavity [2]. In recent years, dental procedures such as the number of fillings, root canals, crowns and extractions that children and adolescents undergo have significantly increased. Dental procedures are generally performed under local anesthesia and most local anesthetics used in dentistry are bitter. Dentists are left with no choice other than injecting bitter drugs like local anesthetics in the oral cavity, which are associated with anxiety in patients. LID injections produce a metallic/bitter aftertaste that can last up to 2 h after the anesthetic disappears [14]. There is a need to develop a better tasting LID injectable that is easy to scale-up and provide local anesthesia without delay. Based on the popularity of LID injection in dental procedures, it is expected that the resulting anesthetics will have a huge potential in dental practices worldwide.

Several approaches such as complexation with hydroxypropyl-β-cyclodextrin [2], the addition of sweeteners [2,15], and coupling of cationic lidocaine with anionic sweeteners like saccarinate and acesulfamate [8] were reported in the literature. A strong interaction between hydroxypropyl-β-cyclodextrin and lidocaine helps in masking the bitter taste of the drug; however, such an inclusion complex might delay the release and availability of the free drug for the onset of anesthetic action [2]. This study aims to assess the ability of a range of flavors along with a sweetener to mask the bitter taste of dental LID injection. Flavors are generally used for oral products such as syrups, chewable tablets, suspensions, or gums in order to mask the bitter taste of drugs. Flavors are mixtures of aroma chemicals that are generally included in food and pharmaceutical products for a specific end-use. As per the Flavor Extracts Manufacturers Association (FEMA), flavors at specific concentrations are safe for ingestion. However, several flavors are included in e-cigarettes even when their toxicity in the lungs upon inhalation remains unclear [16]. We hypothesize that the addition of flavors to the bitter-tasting LID dental injection would significantly improve its taste and change the patient’s perspective towards dental procedures. Nevertheless, flavors have never been used in injectable formulations and their toxicity on a dental tissue remains unknown. In this context, this study intends to look at the toxicity of selected flavors in primary gingival keratinocytes (PGK). Bitter taste masking flavors such as chocolate natural and artificial flavor (CTE), raspberry flavor artificial (RAS), cherry flavor (CHR), bitterness suppressor flavor (BSF), lemon flavor extract (LFE) were employed in this study. Sodium saccharin was added as a sweetener to minimize the metallic/bitter aftertaste of the formulation, leaving behind a lingering sweet taste. In this work, we assessed the cytotoxicity of selected flavors in primary gingival keratinocytes (PGK). Based on the cytotoxicity data, carefully chosen flavors and sweetener were included in Xylocaine^®^ (2%) for taste analysis using an electronic tongue (ETongue).

## 2. Results and Discussion

**Figure 1:**
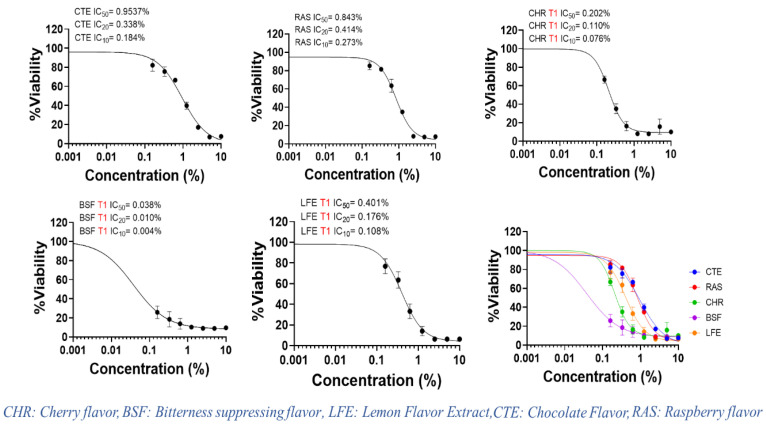
Dose–response curves of selected flavors via MTT proliferation assays in human primary gingival keratinocytes.

We tested the cytotoxic effects of CTE, RAS, CHR, BSF and LFE in PGK using the MTT assay. These flavors were chosen for the study as they are widely used in masking the bitter taste of drugs and especially liked by children [17,18]. The MTT assay is a popular, well accepted in vitro assay to measure cell viability and proliferation. The nicotinamide adenine dinucleotide phosphate NAD(P)H-dependent cellular oxidoreductase enzymes in viable cells are capable of reducing the tetrazolium dye to an insoluble purple-colored formazan, which can be measured spectrophotometrically at 570 nm after dissolution. Cell proliferation of PGK was measured by comparing the purple color formation under the experimental conditions of the study. The reduction in the cell number by flavors can be explained by inhibition of cell proliferation and/or cell killing. The IC_50_ value, the concentration that results in 50% suppression of PGK, was used as a parameter for cytotoxicity in this study [19]. After three hours of treatment, a dose-dependent cell death was induced by all flavors compared to the untreated control. The cytotoxicity of all flavors in PGK increased with concentration compared with the control group. This was further confirmed by morphological analysis by Incucyte S3 live cell imaging system (Essen BioScience, Inc., Ann Arbor, MI, USA). When PGK were exposed to experimental media under control conditions, the cells appeared healthy with a squamous shape [20]. The mean IC_50_, IC_20_ and IC_10_ values of flavors were calculated and presented in Table 1. The mean IC_50_ values of CTE, RAS, CHR, BSF and LFE in PGK were found to be 9.54, 8.43, 2.21, 0.38 and 4.01 mg/mL. Of all the flavors tested, CTE was found to be least toxic, while BSF exhibited the highest toxicity. The dose–response curves of CTE, RAS, CHR, BSF and LFE in PGK are shown in Figure 1.

**Figure 2:**
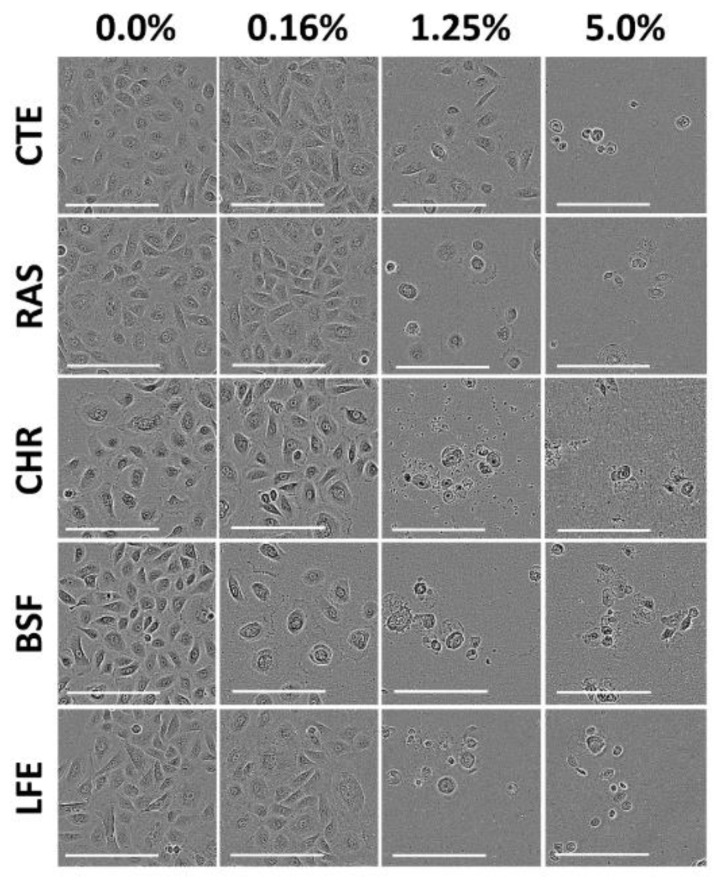
Cytotoxicity assessment of flavors in human primary gingival keratinocytes. Representative image of morphological changes in Keratinocytes (20×) after treatment with either chocolate natural and artificial (CTE), raspberry flavor artificial (RAS), cherry flavor (CHR), bitterness suppressor flavor (BSF) and lemon flavor extract (LFE). Concentrations of flavors used were 0.16%, 1.25%, and 5% *v*/*v* in cell culture media. Images were captured after 72 h of treatment. Scale bars: 200 µm.

Flavors contain a complex chemical composition of excipients such as diluents, antioxidants, defoamers, preservatives, emulsifiers, stabilizers, flavor enhancers, anti-wetting agents and anti-caking agents. The composition of flavors can vary as they are not regulated by the FDA. Agencies such as FEMA and European Food Safety Authority (EFSA) are responsible for approving the chemical formulation of flavors for worldwide use [21]. The toxicity of flavors can be related to their chemical composition. Though it is hard to find the specific chemical composition of flavors from the literature or the product labels, their safety data sheet gives some insights into the chemical composition. CTE mainly contains cocoa extract, vanillin and ethyl vanillin [22]. Both vanillin and ethyl vanillin are GRAS listed and approved for use in nonparenteral formulations. The other components of CTE such as propylene glycol (>50%), corn syrup, benzyl alcohol, glycerin and glucose are also non-toxic in nature. Therefore, the effect of CTE on PGK may be less pronounced. The components of RAS flavor include (*Z*)-hexenol, hexanal, (*E*)-2-hexenal, 2-heptanone, δ-octalactone, δ-decalactone, geraniol, α-ionone, β-ionone, and terpinen-4-ol [23,24]. In addition, RAS also contain excipients such as propylene glycol (>50%) and ethyl alcohol (25–50%). The slightly higher toxicity of RAS compared with CTE could be due to the presence of ethyl alcohol. LFE is mainly composed of ingredients such as ethyl alcohol, pinenes and limonene. The ethyl alcohol concentration in LFE is very high, approximately 78–82%. This could have resulted in the toxicity of LFE. While the composition of BSF remains undisclosed, CHR flavor contained about 30–45% *w*/*w* ethyl alcohol along with propylene glycol and benzaldehyde. Benzaldehyde is known to cause irritation of respiratory airways in animal and occupational exposure studies [25]. The presence of ethyl alcohol along with benzaldehyde in CHR could have caused low cell viability and higher toxicity in comparison with CTE, RAS and LFE. Ethyl alcohol is commonly used in flavors as a solvent and diluent. However, its toxicity is reported in the literature. At concentrations above 0.1–0.5%, ethyl alcohol toxicity was observed in various cell models such as MCF-7, RAW-264.7 and human umbilical vein endothelial cells (HUVEC) [26]. Though cell lines have various degrees of sensitivity to organic solvents, it is highly likely that ethyl alcohol has contributed to significant toxicity of the flavors tested in our study. The morphological analysis corroborates with the results of cytotoxicity of the flavors. Morphological analysis showed marked reduction in cell number for BSF at lower concentration (0.16% *v*/*v*) as shown in Figure 2, while CTE, RAS, CHR and LFE did not show significant toxicity at 0.16% *v*/*v*. However, at higher concentrations of 1.25% *v*/*v* and 5% *v*/*v*, a significant reduction in cells was observed for all flavors.

**Figure 3:**
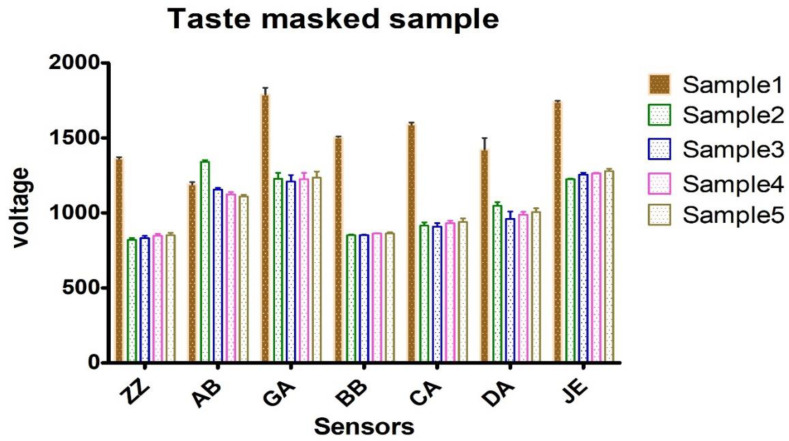
Astree ETongue voltage signals between preparations of sample 1, sample 2, sample 3, sample 4 and sample 5. Sample 1: Xylocaine^®^, Sample 2: Xylocaine^®^ + 0.338% CTE + 0.09% sodium saccharine, Sample 3: Xylocaine^®^ + 0.184% CTE + 0.09% sodium saccharine, Sample 4: Xylocaine^®^ + 0.414% RAS + 0.09% sodium saccharine, Sample 5: Xylocaine^®^ + 0.237% RAS + 0.09% sodium saccharine.

**Figure 4:**
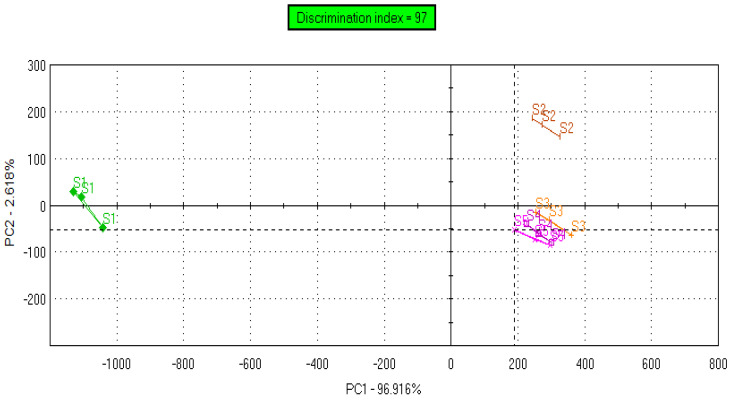
Taste map of formulations based on principal component analysis (PCA) of sample 1 (S1), sample 2 (S2), sample 3 (S3), sample 4 (S4) and sample 5 (S5). S1: Xylocaine^®^, S2: Xylocaine^®^ + 0.338% CTE + 0.09% sodium saccharine, S3: Xylocaine^®^ + 0.184% CTE + 0.09% sodium saccharine, S4: Xylocaine^®^ + 0.414% RAS + 0.09% sodium saccharine, S5: Xylocaine^®^ + 0.237% RAS + 0.09% sodium saccharine.

**Figure 5:**
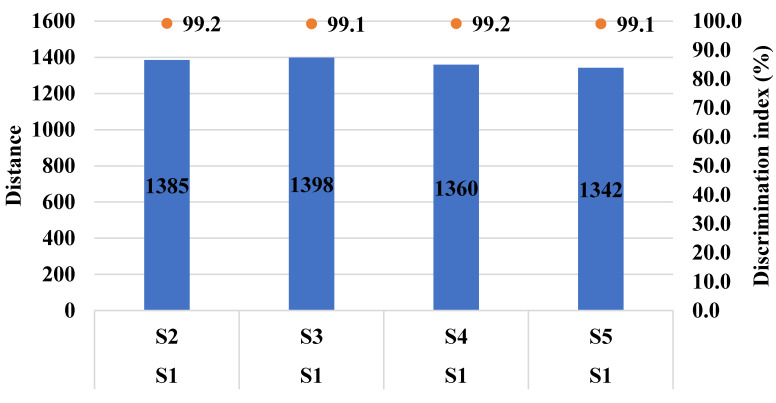
Pairs comparison of samples [S1–S2], [S1–S3], [S1–S4], [S1–S5]. S1: Xylocaine^®^, S2: Xylocaine^®^ + 0.338% CTE + 0.09% sodium saccharine, S3: Xylocaine^®^ + 0.184% CTE + 0.09% sodium saccharine, S4: Xylocaine^®^ + 0.414% RAS + 0.09% sodium saccharine, S5: Xylocaine^®^ + 0.237% RAS + 0.09% sodium saccharine.

**Figure 6:**
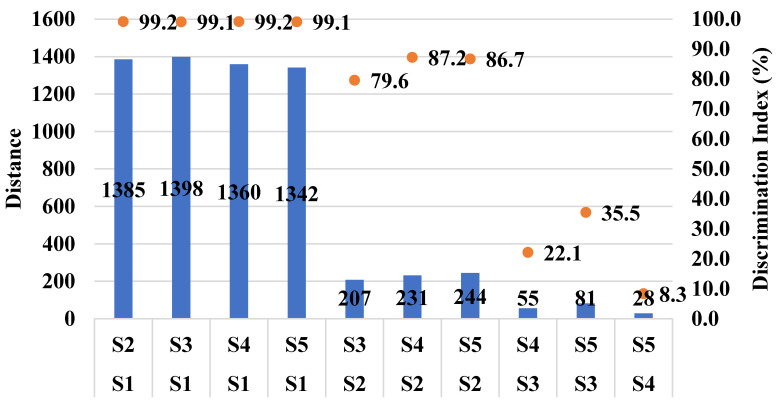
Pairs comparison of the different samples. Sample 1 (S1), sample 2 (S2), sample 3 (S3), sample 4 (S4) and sample 5 (S5). S1: Xylocaine^®^, S2: Xylocaine^®^ + 0.338% CTE + 0.09% sodium saccharine, S3: Xylocaine^®^ + 0.184% CTE + 0.09% sodium saccharine, S4: Xylocaine^®^ + 0.414% RAS + 0.09% sodium saccharine, S5: Xylocaine^®^ + 0.237% RAS + 0.09% sodium saccharine.

Based on the IC50 values determined above in PGK, two non-toxic concentrations (IC20 and IC10) of CTE and RAS were added to Xylocaine^®^ 2% along with 0.09% sodium saccharine for taste analysis using ETongue. With an ETongue, it is possible to investigate the taste of a number of formulations in a rapid manner without any safety concerns that arise from human testing. The signals observed on each of the seven ETongue sensors between the preparations were presented in Figure 3. Signals intensity difference can be observed between the samples regardless of the sensors. The signal of each sensor on each assay was integrated into a matrix of data that was computed by multidimensional statistic tools. A taste map based on PCA was generated using all sensors (Figure 4). Based on the principle of PCA, the samples with high taste correlation form a cluster. The PCA plot shows the formation of three distinct clusters. The data points of Xylocaine^®^ 2% (control) are located on the left side in the middle, while the data points of samples containing flavors (sample 2, sample 3, sample 4 and sample 5) are located on the right side. This indicates a clear difference in taste between the control and test samples. The Euclidian distances between formulations were calculated to assess the taste proximity between samples: the lower the distance, the more similar the taste. The Euclidean distances were calculated from the cluster center where the distance between points within a cluster in minimum, while the distance to points of different clusters is maximum. The control (sample 1) is the most discriminated of all samples. We observed that the first principal component (PC1) showed 96.91% variability between the samples, whereas the second principal component (PC2) showed 2.62% variability between the samples. PC1 can be used to explain the difference between cluster 1 (comprising of sample 1) and other clusters (comprising of sample 2, sample 3, sample 4 and sample 5). In addition, the Euclidean distance of each sample from sample 1 (control) can be used to define the taste differences. From sample 1, the Euclidean distances of sample 2, sample 3, sample 4, sample 5 were 1382, 1398, 1360 and 1342, respectively. This indicates that sample 1, without any flavor or sweetener, has a different taste compared to samples 2, 3, 4 and 5. CTE appeared to be slightly more efficient at masking the LID taste compared with RAS. Similarly, the difference between sample 2 and samples 3, 4 and 5 can be explained by PC2 and the Euclidean distance from sample 2. From sample 2, the Euclidean distances of samples 3, 4, 5 were 207, 231 and 244, respectively. The PC2 accounts for only 2.62% variability. This indicates only a small difference between the taste of sample 2 (Xylocaine^®^ + 0.338% CTE + 0.09% sodium saccharine) compared with samples 3 (Xylocaine^®^ + 0.184% CTE + 0.09% sodium saccharine), 4 (Xylocaine^®^ + 0.414% RAS + 0.09% sodium saccharine), 5 (Xylocaine^®^ + 0.237% RAS + 0.09% sodium saccharine). In addition, a discrimination index (DI in %) was determined for each sample. This indicator takes into account the average difference between the pairs to compare, as well as the dispersion of each sample. The closer the index to 100%, the greater the distance between the centers of gravity and the smaller the dispersion within groups. The DI helps to assess the significance of the difference between the groups. A higher DI number specifies less similarity between samples. The results from Figure 5 showed a clear overall taste difference between the control (S1) and the samples S2, S3, S4 or S5. Regardless of the pairs [S1–S2], [S1–S3], [S1–S4] or [S1–S5], the DI is > 90% and is interpreted as a significant taste difference between the control (S1) and test samples S2, S3, S4 or S5. This higher DI number (>90%) between S1 and test samples S2, S3, S4 or S5 corroborate with distance values. Figure 6 displayed pairs comparison between [S2–S3], [S2–S4], [S2–S5], [S3–S4], [S3–S5] and [S4–S5]. Not much difference in taste was observed with higher concentrations of flavors in samples S2 and S4 as compared to S3 and S5 with a lower concentration of flavors. This indicates reasonable taste-masking can be achieved with CTE and RAS at concentrations of 0.184% and 0.237% *v*/*v*, respectively. Further increase in CTE and RAS concentrations may not significantly improve the formulation taste. The electrochemical taste sensing using an ETongue could provide a useful indication of maximum taste masking efficiency that could be achieved with flavors, which may be helpful in limiting their use in injectables to the required level. Further, the distance between the samples S3 with 0.184% of CTE appeared to have a closer taste to RAS containing S4 and S5 samples than with S2 containing 0.338% of CTE. The repeatability of the measurements on the Astree ETongue was determined for each sample on three replicates (Table 2) and the results were comparable.

This study has some limitations that have to be mentioned. In this study, we have not identified the individual flavoring chemicals present in each flavor, instead, we tested the cytotoxicity of flavors as a whole, following appropriate dilution in the cell culture medium. Further, our study did not assess the in vitro toxicological effects of flavors such as metabolic activity and release of inflammatory mediators in PGK. In the study, we used flavors in the liquid form as they can be easily mixed with an injectable product for taste analysis. Though pure solid flavors would give a better idea in terms of toxicity, they might pose a solubility issue in the injectable formulation. For this reason, we opted for liquid flavors rather than pure solid flavors. In addition, the stability of LID and epinephrine in the presence of flavors remains to be studied. Based on the data, we plan to use pure solid flavors in our future studies for the development and characterization of a better tasting LID injectable formulation, including the formulation stability. The use of pure solid flavors will eliminate the toxicity resulting from excipients used in the preparation of liquid flavors. Despite these limitations, the present study indicates the possibility of improving the taste of lidocaine dental injection through inclusion of sodium saccharin and flavors such as CTE and RAS at 0.184% and 0.237% *v*/*v*, respectively.

## 3. Materials and Methods

### 3.1. Materials

Sodium saccharin (USP, 99%) was purchased from Acros Organics (Fair Lawn, NJ, USA). Flavors such as chocolate natural and artificial flavor (Lot no: 4HK0036), raspberry flavor artificial (Lot no: 4HI0016), cherry flavor artificial/natural (Lot no: 4HE0039), bitterness suppressor flavor (Lot no: 4HH0011), lemon flavor extract (Lot no: 2HK0135) were procured from Spectrum Pharmacy Products (New Brunswick, NJ, USA). Keratinocyte Growth Kit, Primary Gingival Keratinocytes (PCS-200-014™, Normal, Human), Dermal Cell Basal Medium were procured from ATCC (Manassas, VA, USA). Dish Easy Grip (60 × 15 MM) was procured from VWR (Radnor, PA, USA). Pipet Sterile 10 mL was procured from Fisher Scientific (Waltham, MA, USA), Thiazolyl Blue Tetrazolium Bromide (MTT) was procured from GoldBio (Saint Louis, MO, USA). Xylocaine^®^ with epinephrine injectable 2% was procured from Keeler Ophthalmic Instruments (Malvern, PA, USA). All solvents used were of analytical grade. Deionized water was used throughout the experiments.

### 3.2. Cytotoxicity of Selected Flavors in Primary Gingival Keratinocytes (PGK)

The PGK were grown as adherent monolayers in flasks with dermal cell basal medium, supplemented with 10% fetal bovine serum (FBS) and 1% penicillin and streptomycin in a humidified incubator with 5% CO_2_ at 37 °C. Cell viability was performed using 3-(4,5-dimethylthiazol-2-yl)-2,5-diphenyltetrazolium bromide (MTT) assay to determine the cytotoxicity of 5 flavors: CTE, RAS, CHR, BSF and LFE. Briefly, in a 96-well flat-bottom cell culture plate, cells were seeded at a density of 8 × 10^3^ cells/well (180 µL in each well) and incubated for 24 h. Serial dilutions (0.16–10%) of flavors were made in culture medium and arranged in 96-well plates with a negative control (culture medium only). After 72 h, 20 µL of 5 mg/mL of MTT was added to the plate and incubated for 3 h at 37 °C. Following incubation, the supernatant was removed, insoluble formazan precipitates were dissolved in 150 µL of dimethyl sulfoxide (DMSO). Subsequently, the plate was shaken for 5 min and the absorbance was measured at 570 nm using a SpectraMax i D3 Multi-Mode Microplate Reader (Molecular Devices, Sunnyvale, CA, USA). The MTT assay was performed in technical triplicates for each flavor and repeated 3 separate times. Data were normalized by setting treatment wells as percentages of the negative control (100%). Graphs were plotted using the GraphPad Software (GraphPad, Prism 8, San Diego, CA, USA). The GraphPad Prism software was also used to compute IC_50_, IC_20_, and IC_10_ values with the log inhibition vs. normalized response-variable slope with the top and bottom constraints set to 100% and 0%, respectively. The results were based on technical triplicates [27,28]. The morphological analysis was confirmed using Incucyte S3 live cell imaging system (Essen BioScience, Inc., Ann Arbor, MI, USA).

### 3.3. Preparation of Samples for Taste Analysis

Xylocaine^®^ (2%) with epinephrine injection was used for preparing test samples. Test samples were prepared by mixing Xylocaine^®^ (2%) with 0.09% *w*/*v* sodium saccharine and selected flavors such as CTE and RAS as shown in Table 3.

### 3.4. Taste Analysis Using Astree Electronic Tongue (ETongue)

The assays were realized on Astree ETongue system equipped with an Alpha M.O.S. sensor set #2 (for pharmaceutical analysis) composed of 7 specific sensors (ZZ, AB, GA, BB, CA, DA, JE). The 48-positions autosampler and 25 mL-beakers were used for sampling. Acquisition times were fixed at 120 s. All the data generated on Astree system were treated using multidimensional statistics on AlphaSoft V2020 software. Prior to the analysis of the products, the sensors were conditioned in HCl 0.01 M for 6 h; calibrated in the HCl 0.01 M for 1 h. A diagnostic was done in HCl 0.01 M, NaCl 0.01 M and monosodium glutamate 0.01 M to evaluate the performance of the sensors. A sample volume of 25 mL and acquisition time of 120 s was set with 180 s analysis time per sample analysis. The ETongue signal in each solution was measured at the equilibrium on 7 sensors (average between 100 and 120 s). Three replicates were taken into account for the analysis. Sensors were cleaned up 2 times with deionized water between each measurement from one sample to another in order to avoid cross-contamination. After the analysis, an extra diagnostic was conducted for the follow up on sensor performance. The signal of each sensor on each assay was integrated into a matrix of data that was computed by multidimensional statistic tools.

### 3.5. Data Analysis

For taste assessment, the sensor signals of samples 2, 3, 4 and 5 were compared with sample 1 (control, Xylocaine^®^) using principal component analysis (PCA). A taste map based on PCA was generated using all sensors. PCA comprehends the multidimensional data by converting sensor signals to principal components in a new space with a two-dimensional graph. The discriminating factors are principal component 1 (PC1) on the *x*-axis and principal component 2 (PC2) on the *y*-axis. Using the PCA map, samples were visually evaluated based on the location of the measured samples. Since Xylocaine^®^ dental injection (sample 1) exhibits a known unpleasant bitter taste, the more the distance between sample 1 and other samples (2, 3, 4, 5), the greater is the difference in taste. Each sample was analyzed three times and presented as a triangle on the taste map. The distance between two samples represented the Euclidean distance between their corresponding triangles [2].

## 4. Conclusions

Local anesthetic injections in dentistry are a common source of anxiety and fear during dental procedures. In this study, we used a combination of flavor and sweetener to improve the taste of LID dental injection. CTE and RAS were found to be less toxic in PGK with IC_50_ values of 9.54 and 8.43 mg/mL, respectively. Incorporation of sodium saccharine and CTE or RAS in Xylocaine^®^ 2% injection showed a clear difference in taste using Astree ETongue. The distances between control (S1) and the samples S2, S3, S4 or S5 were significant with the DI > 90%, suggesting a significantly different taste difference with the addition of sodium saccharine and flavors. These results suggest that better tasting LID dental injection could be achieved through the addition of sodium saccharin and flavors such as CTE and RAS. Further, the long-term toxicity of flavors should be established in other cell culture and animal models. Our future studies will investigate pure solid flavors with no additives for suitability as parenteral additives.

## Figures and Tables

**Table 1:** The inhibitory concentration (IC_50_, IC_20_, IC_10_) values of selected flavors. Values are presented as mean (*n* = 3).

Flavors	IC_50_ (mg/mL)	IC_20_ (mg/mL)	IC_10_ (mg/mL)
Chocolate flavor (CTE)	9.54	3.38	1.84
Raspberry flavor (RAS)	8.43	4.14	2.37
Cherry flavor (CHR)	2.21	0.86	0.49
Bitter suppressing flavor (BSF)	0.38	0.1	0.04
Lemon flavor (LFE)	4.01	1.76	1.08

**Table 2:** Mean standard deviation (SD) and relative standard deviation (RSD) for each sample.

Sample Name	Code	SD	%RSD
Sample 1 (control)	S1	20.1	1.3
Sample 2	S2	14.1	1.3
Sample 3	S3	17.9	1.7
Sample 4	S4	13.0	1.2
Sample 5	S5	17.0	1.6

**Table 3:** Composition of various test samples for taste analysis using electronic tongue (ETongue).

Sample Number	Lidocaine HCl/Epinephrine 2%	Sodium Saccharine (% *w*/*v*)	Chocolate Natural and Artificial Flavor (% *v*/*v*)	Raspberry Flavor Artificial (% *v*/*v*)
Sample 1 (control)	Lidocaine/Epinephrine Injection 2%	-	-	-
Sample 2	Lidocaine/Epinephrine Injection 2%	0.09	0.338	-
Sample 3	Lidocaine/Epinephrine Injection 2%	0.09	0.184	
Sample 4	Lidocaine/Epinephrine Injection 2%	0.09	-	0.414
Sample 5	Lidocaine/Epinephrine Injection 2%	0.09	-	0.237
